# Correlates of post-traumatic growth among persons bereaved from cancer: A systematic review and meta-analysis

**DOI:** 10.1371/journal.pone.0300291

**Published:** 2024-03-15

**Authors:** N. V. Sri Lakshmi K., Eslavath Rajkumar, Aswathy Gopi, P. V. Hareesh, John Romate, R. Lakshmi, John Abraham

**Affiliations:** 1 Department of Psychology, Central University of Karnataka, Kalaburagi, Karnataka, India; 2 Department of Liberal Arts, Indian Institute of Technology Bhilai, Durg, Chhattisgarh, India; 3 Department of Applied Psychology, Central University of Tamil Nadu, Thiruvarur, Tamil Nadu, India; 4 St. John’s Medical College, St. John’s National Academy of Health Sciences, Bangalore, India; The Hong Kong Polytechnic University, HONG KONG

## Abstract

**Background:**

Recent research identified that cancer bereavement can lead to post-traumatic growth (PTG). Although PTG and its correlates are well explored in cancer patients and survivors, persons bereaved from cancer have received scant attention. Therefore, the present review attempts to identify the correlates of PTG among persons bereaved from cancer.

**Methods:**

A systematic search in PubMed, Web of Science, APA PsycNet, Science Direct, Scopus, and Wiley was conducted to identify quantitative studies published in English, resulting in 12 eligible reports being included in the final analysis. JBI critical checklists were employed to appraise the risk of bias.

**Results:**

The review identified 17 correlates, which were classified into four categories: demographic factors (age, gender, religious status, level of education), loss-related factors (time since death, quality of death, prolonged grief symptoms), interpersonal factors (relationship to the deceased, social support, attachment style, bereavement behaviours) and intrapersonal factors (resilience, coping, rumination, benevolence, meaningfulness, self-worth). Random effects meta-analyses on six correlates revealed correlation coefficients of age = -0.02 (95% CI: -0.35–0.31), gender = 0.27 (95% CI: 0.08–0.45), time since death = 0.09 (95% CI: -0.02–0.20), quality of death = 0.29 (95% CI: -0.01–0.54), prolonged grief symptoms = 0.22 (95% CI: 0.08–0.35) and relationship to the deceased = 0.13 (95% CI: -0.03–0.29). Fixed effects meta-analysis was performed for social support (r = 0.13, 95% CI: 0.04–0.21). However, PTG was found to be significantly associated with gender, prolonged grief symptoms, and social support.

**Conclusions:**

Very few studies examined PTG among persons bereaved from cancer, highlighting the need for increased attention, understanding, and conceptualisation of PTG in the population.

## Introduction

Cancer is one of the leading causes of mortality worldwide, with an estimated 10 million deaths in 2020 alone, leaving behind their families and friends to cope with a traumatic loss [1]. Cancer deaths are estimated to reach 16.2 million by 2040 [2]. Both the final stage of a relative’s cancer (anticipatory grief) and the period that follows the loss (grief and bereavement) are marked for the family by a heavy burden owing to adjustments in how the family functions, shifts in roles, changes to interpersonal relationships, and other issues the family may deal with [3]. Therefore, as part of palliative care, it is crucial to comprehend the agony felt by family members of cancer patients as well as the aftereffects of bereavement.

Informal caregivers are individuals who have a significant personal relationship with and provide a broad range of unpaid assistance to a person with a chronic or disabling condition outside of a professional or formal framework [4]. Through the term, ‘*persons bereaved from cancer*’, the present study includes caregivers, family members, friends and relatives who lost a loved one to cancer. Since each individual adjusts to loss differently, many studies have focused on the factors contributing to poor psychological adjustment to bereavement [5]. Earlier studies revealed that family members of cancer patients are affected by negative experiences like long-term grief [6], complicated grief [7], bereavement maladjustment [8], loneliness, sleep disturbance [9], depression [10], and intrusive difficult images of the deathbed [11]. However, cancer recovery may be considered a psychosocial transformation with positive and negative potentials [12]. The concept of post-traumatic growth (PTG) was pioneered by Tedeschi and Calhoun [13] to describe the phenomena of positive psychological change to cope with highly stressful and challenging life situations. PTG refers to the development of adaptive functioning greater than the degree the individual manifested before the stressor occurred [14]. In bereaved people, PTG helps improve the quality of their relationships, outlook on life, perception of personal strength, enjoyment of life, and spirituality [13, 15]. It is to be noted that previous research also suggests that PTG can be influenced by cultural factors, either through affecting the fundamental world-view assumptions of individuals, and type of social support they receive [16] or in terms of how PTG is expressed and experienced [17].

Nonetheless, to date, literature has largely focused on PTG in cancer patients [18, 19], and only a few have investigated PTG in caregivers [20, 21]. Furthermore, available reviews synthesised data on the correlates of PTG in cancer patients [22], survivors [23] and caregivers [24]. These reviews identified various correlates influencing PTG, such as age at diagnosis [22, 23], age of the bereaved [25–28], gender [22, 25, 28, 29], mental health disturbances like depression, anxiety [22], social support [22, 27–29], optimism [22, 28, 29], cognitive processing [22, 29], spirituality and religious coping [22, 27, 28], relationship to the deceased [22, 25, 26], time since loss [25–28], and coping strategies [28, 29] across various populations such as bereaved, cancer survivors, older adults, adolescents etc. Importantly, they indicated that different correlates influence PTG in different populations. Thus, specifically understanding the correlates of PTG in persons bereaved from cancer becomes warranted. By synthesising data from diverse sources, a meta-analysis would enhance the precision and reliability of the findings, offering a nuanced perspective on the correlates of PTG. It allows the future research to decide which correlates of PTG need to be targeted and prioritised when designing interventions. The meta-analysis not only enhances the impact of existing research but also contributes to evidence-based decision-making in a more compelling and informative manner. Therefore, the current review and meta-analysis aimed to examine the available literature on the various correlates of PTG among persons bereaved from cancer.

According to Calhoun and Tedeschi [30], PTG is greatly influenced by demographic, situational, interpersonal, and intrapersonal factors. Hence, the correlates identified in this review are categorised into the same. Additionally, the findings could present research gaps in the study area and inform rigorous studies in the future. The results would enlighten and guide corresponding authorities and healthcare practitioners on acceptable and feasible therapies, interventions, and support for persons who have lost a loved one to cancer.

## Method

The study was conducted according to the Preferred Reporting Items for Systematic Reviews and Meta-Analyses (PRISMA) [31]. The protocol is registered in and can be accessed through PROSPERO, with the ID: CRD42022322775.

### Eligibility criteria

Quantitative studies (cross-sectional and longitudinal studies) that report at least one correlate of PTG among persons bereaved from cancer were included. Due to the small number of studies that directly reported correlation coefficients between different correlates and PTG, studies that presented data related to the correlates in the form of means, and standard deviations or t-values from independent sample t-tests or beta coefficients from regression models were also included. These measures are then used to calculate the correlation between the correlates and PTG either by direct calculation or through conversions. The conversions were made wherever appropriate after careful consideration by the first two authors (SL and RE). Reports published only in English were included. There was no limit set on the year of publication, to ensure that all relevant studies published to date were included. Grey literature and secondary data analysis studies were excluded from the review as their quality is sometimes questionable. Conference abstracts, intervention studies, case studies, purely qualitative studies, conceptual papers proposing models or theories related to PTG, commentaries, reviews, and books were excluded.

### Information sources

A systematic search was conducted in databases: PubMed, APA PsycNet, Science Direct, Scopus, Web of Science, and Wiley to identify studies on correlates of PTG in persons bereaved from cancer till August 2022. The searches were re-run, and relevant studies were retrieved for inclusion before the analysis. Reference lists of the pertinent articles facilitated the search for additional papers that satisfied the inclusion criteria.

### Search strategy

The following search strategy was adapted for the databases: (correlates OR predictors OR factors) AND (post-traumatic growth OR posttraumatic growth OR post traumatic growth) AND (bereaved OR grief OR mourning OR family) AND (cancer OR death by cancer). The systematic search was limited to peer-reviewed journals without confines to study location or year of publication. Table 1 shows the detailed search strategy used for various databases.

**Table 1:** Search strategy followed for various databases and articles retrieved.

Databases	Search strategy	Articles found
PUBMED	(Correlates OR predictors OR factors) AND (posttraumatic growth OR post-traumatic growth OR post traumatic growth) AND (bereaved OR grief OR mourning OR family) AND (cancer OR death by cancer)	101
WEB OF SCIENCE	(((((((((((TI = (Correlates)) OR TI = (factors)) OR TI = (predictors)) AND TI = (post traumatic growth)) OR TI = (post-traumatic growth)) OR TI = (posttraumatic growth)) AND TI = (bereaved)) OR TI = (mourning)) OR TI = (family)) AND TI = (cancer)) OR TI = (death by cancer)) NOT TI = (cancer survivors) and Articles (Document Types)	5806
SCIENCE DIRECT	(Correlates OR predictors OR factors) AND (posttraumatic growth OR post-traumatic growth OR post traumatic growth) AND (bereaved OR grief OR mourning OR family) AND (cancer OR death by cancer)	2631
APA PSYCHNET	Title: post-traumatic growth OR Title: post traumatic growth OR Title: posttraumatic growth AND Title: bereaved OR Title: mourning OR Title: family AND Title: cancer OR Title: death by cancer NOT Title: cancer survivors	53
WILEY	"(Correlates OR predictors OR factors) AND (posttraumatic growth OR post-traumatic growth OR post traumatic growth) AND (bereaved OR grief OR mourning OR family) AND (cancer OR death by cancer) NOT (Cancer Survivors)" anywhere	326

### Selection process

The first two authors (SL and ER) did the study conceptualisation and independently searched for relevant records in databases. All the search results were exported to Zotero. Post removal of duplicates, the data was saved to MS Excel for screening. Two reviewers (SL and AG) independently identified records that met the eligibility criteria through title and abstract screening, followed by full-text screening. Reasons for exclusion were documented at each stage of screening. Disagreements regarding the selection of studies were resolved through frequent discussions and, when needed, by consulting a third reviewer (ER). Due to the small number of studies left after the full-text analysis; a reviewer (SL) conducted a manual search.

### Data collection process

Two authors (SL and HPV) independently extracted relevant information from the finalised articles. Disagreements were handled by debate and consensus. In case of conflicts, a third reviewer (ER) was approached. Consequently, study characteristics such as the name of the author(s), study design, year of publication, study location, sample age, sample size, time since death, population, correlates, and effect size for the relationship with variables were extracted.

### Study risk of bias assessment

Three reviewers (SL, AG, HPV) examined the risk of bias using standardised critical appraisal tools. The JBI critical appraisal checklist for analytical cross-sectional studies and the JBI critical appraisal checklist for cohort studies were used. The evaluation criteria for the JBI checklist for analytical cross-sectional studies were: 1) clear inclusion criteria of the sample, 2) sufficiency of the description of the study subjects and settings, 3) validity and reliability of the measurement of exposure, 4) the presence of objective criteria for measurement of the condition, 5) identification of confounding factors, 6) strategies to deal with the confounding factors, 7) validity and reliability of the outcome measurement, and 8) appropriateness of the statistical analysis. The evaluation criteria for the JBI checklist for cohort studies were: 1) similarity between the two groups, 2) appropriateness of the measurement of the exposure to assign people to groups, 3) validity and reliability of the measurement of the exposure, 4) identification of confounding factors, 5) strategies to deal with the confounding factors, 6) absence of the outcomes of interest at the start of the study, 7) validity and reliability of the outcome measurement, 8) appropriateness of the duration of the follow-up, 9) completeness of the follow-up, 10) strategies to address incomplete follow-up, 11) appropriateness of the statistical analysis. Each item was scored in the following way: yes = 1; no, unclear or not applicable = 0. Discussion among the reviewers and consultation with a fourth reviewer (RJ) helped to resolve disagreements and reach a consensus.

### Effect measures

The effect size index was calculated using the correlation coefficient *r* [with a 95% confidence interval (CI)]. When *r* is not available, other statistics (standardised beta coefficients, t-values) were used to calculate the effect size using the following formulas:

r=β+.05λ,

where λ = 1 when β is nonnegative and 0 when β is negative [32].

$$r=\sqrt{\frac{t^{2}}{t^{2}+df}},$$

where t is the t value obtained from the study and df is the degrees of freedom from the t-test.

### Synthesis methods

Meta-analyses were used to establish a pooled correlation estimate between each variable/correlate and levels of PTG in persons bereaved from cancer; when data from at least two trials was available. For each study, the correlation coefficient was changed to the Fisher’s z scale, and the corresponding variance, standard error, was also calculated using formulas from Borenstein [33].


$$Fisher^{\prime }s\;z=0.5\;\times \;ln\;ln\;\frac{1+r}{1-r}\;;$$

$${\text{V}}_{\text{Z}}=\frac{1}{n-3};$$

$$\text{SE}=\sqrt{V_{z}}$$

These transformed data were used in all analyses. Weighted averages of the transformed scores were calculated using the inverse variance method. The “metan” command of the STATA software (Version 17.0) was used. For reporting, the pooled Fisher’s z was converted back to correlations, along with its confidence intervals, using,

$$\text{r}=\frac{e^{\left(2\;\times \;z\right)}-1}{e^{\left(2\;\times \;z\right)}+1};$$

$$LL_{r}\;=\frac{e^{\left(2\;\times \;LL_{z}\right)}-1}{e^{\left(2\;\times \;LL_{z}\right)}+1};$$

$$UL_{r}\;=\frac{e^{\left(2\;\times \;UL_{z}\right)}-1}{e^{\left(2\;\times \;UL_{z}\right)}+1}.$$

The pooled correlation, with 95% CI: Lower Limit-Upper Limit (CI: LL-UL), was reported. The interpretation of the effect size correlations was done using Cohen’s guidelines: r ≤ .10—very weak effect size; 0.1 ≤ r < 0.3 –weak, 0.3 ≤ r < 0.5 –Moderate, r ≥ 0.5 –strong.

The I^2^ statistic was used to examine heterogeneity. The greater the heterogeneity between the studies, the higher the I^2^ value/percentage. If I^2^ is 50%, a fixed effects model was used; if I^2^>50%, a random effects model was employed. Only results with a p<0.05 were considered statistically significant. The current review initially planned to investigate heterogeneity through subgroup analyses and meta-regression, but they could not be performed due to insufficient papers and/or data. All analyses were conducted in STATA 17.

### Reporting bias assessment

Publication bias occurs when the studies that are part of the analysis systematically deviate from all the research that ought to be part of the analysis. Studies with larger-than-average effects are generally more likely to be published, which may cause the overall effect to shift upward [33]. This bias may cause an overestimation of the true effect size and compromise the validity of the meta-analysis [34]. To address this issue, funnel plot was used to determine the publication bias of included studies, where the gaps or asymmetries in the plot reveal potential publication bias. Egger’s test was performed to examine the small study effect; a pattern where the effect size is larger in small studies compared to large studies [35]. Small-study effects usually are a result of publication bias, outcome reporting bias and heterogeneity [36]. So, Egger’s test is used to confirm the presence of publication bias; with the results showing publication bias when p<0.05.

## Results

### Study selection

**Fig 1:**
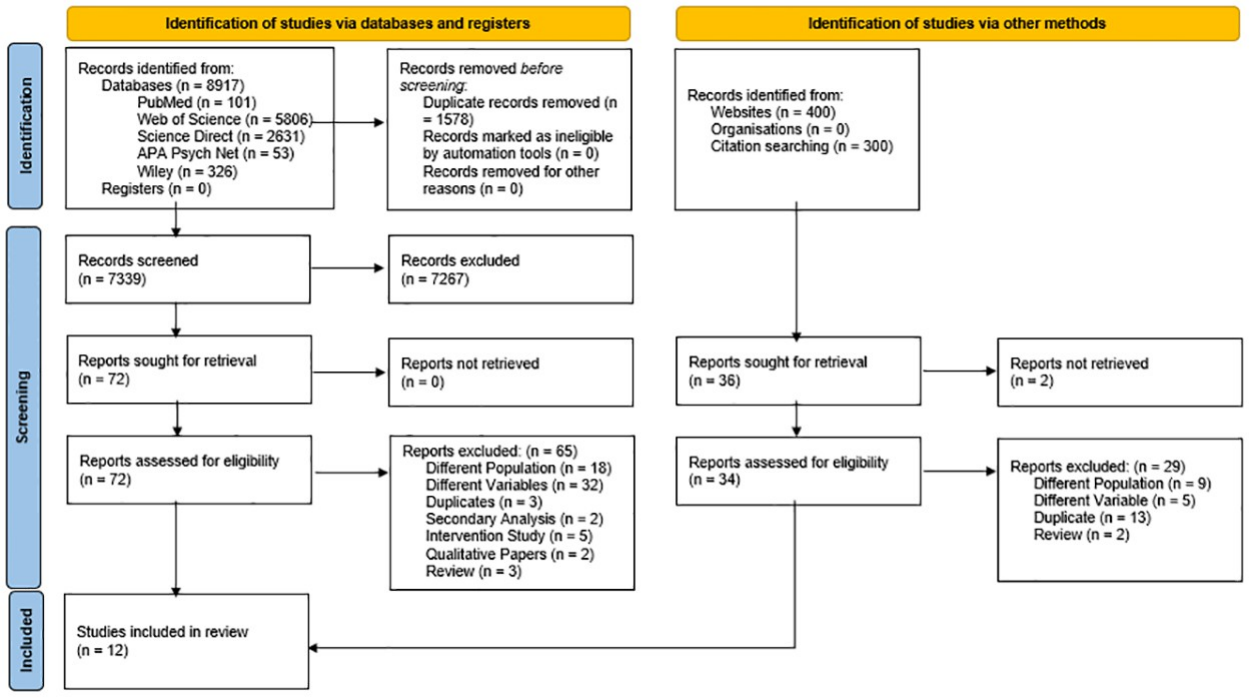
PRISMA flow diagram showing the study selection process.

The initial search on databases using keywords yielded 8917 records. After removing 1578 duplicates, title/abstract screening was done for the remaining 7339 articles. Consequently, 72 records that met the eligibility criteria were selected for full-text screening of which, 65 reports were removed, including strictly qualitative papers, duplicates, studies with different populations, variables, and secondary analyses. Five more papers that met the inclusion criteria were identified through a manual search. Thus, 12 studies that reported correlates of PTG among persons bereaved from cancer along with results yielded by citation searching were included. Fig 1 shows the PRISMA flow diagram.

### Study characteristics

The 12 included studies provided information about various correlates of PTG among persons bereaved from cancer for a total sample of 3688 participants. Three studies reported correlates of PTG among adolescents who lost a parent [37, 38] or grandparent [39]. Eight studies reported the correlates of PTG in bereaved family caregivers of cancer patients [40–47] with one study specifically looking at bereaved parents [48]. All 12 studies used convenient sampling. Out of the 12 included studies, two were cohort studies [44, 45] and the remaining were cross-sectional studies. All the study samples included a higher number of female participants. The time since death varied between 3 months [40] and ten years [38, 48].

Five papers investigated factors influencing specific dimensions of PTG [39, 43–45, 47]. All the studies used the Post Traumatic Growth Inventory (PTGI) [49] or its translated versions for the assessment of PTG. Table 2 depicts the characteristics of the 12 included studies.

**Table 2:** Characteristics of the studies included (n = 12).

Study	Country	Sample age in years (mean)	Sample size	Time since loss (mean)	Sample	Correlated to PTG	Findings related to dimensions of PTG	Risk of bias
*Cross-sectional studies (n = 10)*								
Currier et al. (2012) [40]	USA	18–56 (21.34)	671	3 to 24 months (10.46 months)	Bereaved family members	Gender, Age, Family education, Relationship to deceased, Time since loss, Self-worth, Benevolence, PG symptoms, Meaningfulness	-	Low
Hatano et al. (2015) [41]	Japan	21–88 (58.2)	35	6 to 8.5 months (7.16 months)	Bereaved caregivers	PTG, Quality of death	-	Moderate
Xu et al. (2015) [47]	China	≥ 18 (39.53)	240	(7.6 years)	Bereaved family	Attachment avoidance, Attachment anxiety, Grief	Significant relation between grief and spiritual change; Attachment avoidance and appreciation of life and personal strength.	Moderate
Hirooka, Fukahori, Akita, et al. (2017) [37]	Japan	15–23 (19.3)	57	Up to 5 years (2.8 years)	Parentally bereaved adolescents	Gender, Age, Social support, Bereavement Behaviours	-	Moderate
Hirooka, Fukahori, Ozawa, et al. (2017) [39]	Japan	15–23 (18.99)	124	Up to 5 years (2.8 years)	Bereaved adolescents	Gender, Age, Relationship to deceased, Grief reactions	Parentally bereaved adolescents scored higher in New Possibilities and Personal Strength than those who lost a grandparent.	Moderate
Hirooka, Fukahori, Taku, et al. (2017) [42]	Japan	≥ 20 (63.0)	805	Up to 5 years (2.6 years)	Bereaved relatives	Gender, Religious beliefs, Quality of death, Intrusive rumination soon after death, Recent intrusive rumination, Deliberate rumination after the loss, Recent deliberate rumination	-	Moderate
Hirooka et al. (2018) [43]	Japan	≥ 20 (63.1)	849	Up to 5 years (2.6 years)	Bereaved relatives	Gender, religious beliefs, relationship to the deceased	Being female and having religious beliefs is associated with greater levels of PTG domains. Older family members and closer relations to the deceased report greater Spiritual Change. Time since death is significantly related to Personal Strength.	Low
Donovan et al. (2021) [48]	Australia	31–67 (47.2)	119	6 months to 10 years (5.6 years)	Bereaved parents	Gender, Education level, Religiousness	-	Moderate
Morris et al. (2022) [38]	Australia	≥ 18	57	10 years (5.5 years)	Parentally bereaved children	Coping	-	Low
Takedomi et al. (2021) [46]	Japan	≥ 20 (61.4)	448	6 months to 6 years	Bereaved families	Age, Gender, Emotion-focused coping, Emotional support, Relationship to the deceased, Problem-focused coping	-	Low
*Cohort studies (n = 2)*								
Lee et al. (2016) [44]	South Korea	(52.3)	25	-	Bereaved family	Resilience at EOL	People with higher levels of family functionality, perceived social support, quality of life, and lower levels of emotional distress when providing care at EOL had higher new possibilities scores.	Moderate
Shimizu et al. (2022) [45]	Japan	20 or above (63.4)	71	(379 days)	Bereaved caregivers	Resilience	Pre‐loss resilience is not associated with any domains of PTG.	Moderate

PTG = Post-traumatic growth; EOL = End of Life; QOL = Quality of Life; PG = Prolonged Grief

### Risk of bias in studies

JBI critical appraisal checklists were used to evaluate risk of bias. Refer to Tables 3 and 4 for the results of the risk of bias assessment. Among the cross-sectional studies, six studies were found to have a moderate risk of bias/moderate quality [37, 39, 41, 42, 47, 48] while the rest four studies had a low risk of bias/high quality [38, 40, 43, 46]. Both the cohort studies have a moderate risk of bias/moderate quality [44, 45]. Most studies did not meet two parameters: (1) identification of confounding factors; and (2) strategies for dealing with confounding variables. A confounding variable is an uncontrolled extraneous variable that has influenced the outcome of the study in an unexpected way [50], by obscuring or altering the effect of the independent variable on the dependent variable [51]. Therefore, as the studies did not state clearly whether they identified confounding variables or how they dealt with them effectively, the true relationship between the various correlates and PTG must be interpreted with caution.

**Table 3:** Results of risk of bias assessment for cross-sectional studies using JBI checklist for analytical cross-sectional studies.

First Author, year	Item number								Total Score	Risk of Bias
	1	2	3	4	5	6	7	8		
Currier et al. (2012) [40]	Y	Y	Y	Y	Y	U	Y	Y	7	Low
Hatano et al. (2015) [41]	Y	Y	Y	Y	N	N	Y	Y	6	Moderate
Xu et al. (2015) [47]	Y	Y	Y	Y	U	N	Y	Y	6	Moderate
Hirooka, Fukahori, Akita, et al. (2017) [37]	Y	Y	Y	Y	U	N	Y	Y	6	Moderate
Hirooka, Fukahori, Ozawa, et al. (2017) [39]	Y	Y	Y	Y	U	N	Y	Y	6	Moderate
Hirooka, Fukahori, Taku, et al. (2017) [42]	Y	Y	Y	Y	U	N	Y	Y	6	Moderate
Hirooka et al. (2018) [43]	Y	Y	Y	Y	Y	N	Y	Y	7	Low
Donovan et al. (2021) [48]	Y	Y	Y	Y	U	N	Y	Y	6	Moderate
Morris et al. (2019) [38]	Y	Y	Y	Y	Y	N	Y	Y	7	Low
Takedomi et al. (2021) [46]	Y	Y	Y	Y	Y	U	Y	Y	7	Low

Note. Item number refers to the items in the JBI Checklist for Analytical Cross-Sectional Studies: 1) clear inclusion criteria of the sample, 2) sufficiency of description of the study subjects and settings, 3) validity and reliability of the measurement of exposure, 4) the presence of objective criteria for measurement of the condition, 5) identification of confounding factors, 6) strategies to deal with the confounding factors, 7) validity and reliability of the outcome measurement, and 8) appropriateness of the statistical analysis

Abbreviations: Y = Yes; N = No; U = Unclear

^a^ Score is awarded as follows: Y = 1; N, U = 0

**Table 4:** Results of risk of bias assessment for cohort studies using JBI checklist for cohort Studies.

First Author, year	Item number											Total Score	Risk of Bias
	1	2	3	4	5	6	7	8	9	10	11		
Lee et al. (2016) [44]	Y	Y	Y	U	U	Y	Y	Y	N	Y	Y	8	Moderate
Shimizu et al. (2022) [45]	Y	Y	Y	U	U	Y	Y	Y	N	Y	Y	8	Moderate

Note. Item number refers to the items in the JBI Checklist for Cohort Studies: 1) similarity between the two groups, 2) appropriateness of the measurement of the exposure to assign people to groups, 3) validity and reliability of the measurement of the exposure, 4) identification of confounding factors, 5) strategies to deal with the confounding factors, 6) absence of the outcomes of interest at the start of the study, 7) validity and reliability of the outcome measurement, 8) appropriateness of the duration of the follow-up, 9) completeness of the follow-up, 10) strategies to address incomplete follow-up, 11) appropriateness of the statistical analysis.

Abbreviations: Y, Yes; N, No; U, Unclear

^a^ Score is awarded as follows: Y = 1; N, U = 0

Moreover, all the studies used non-probability sampling techniques which may not provide a representative sample. Thus, caution must be taken before generalising the current findings to the entire population.

### Publication bias

**Fig 2:**
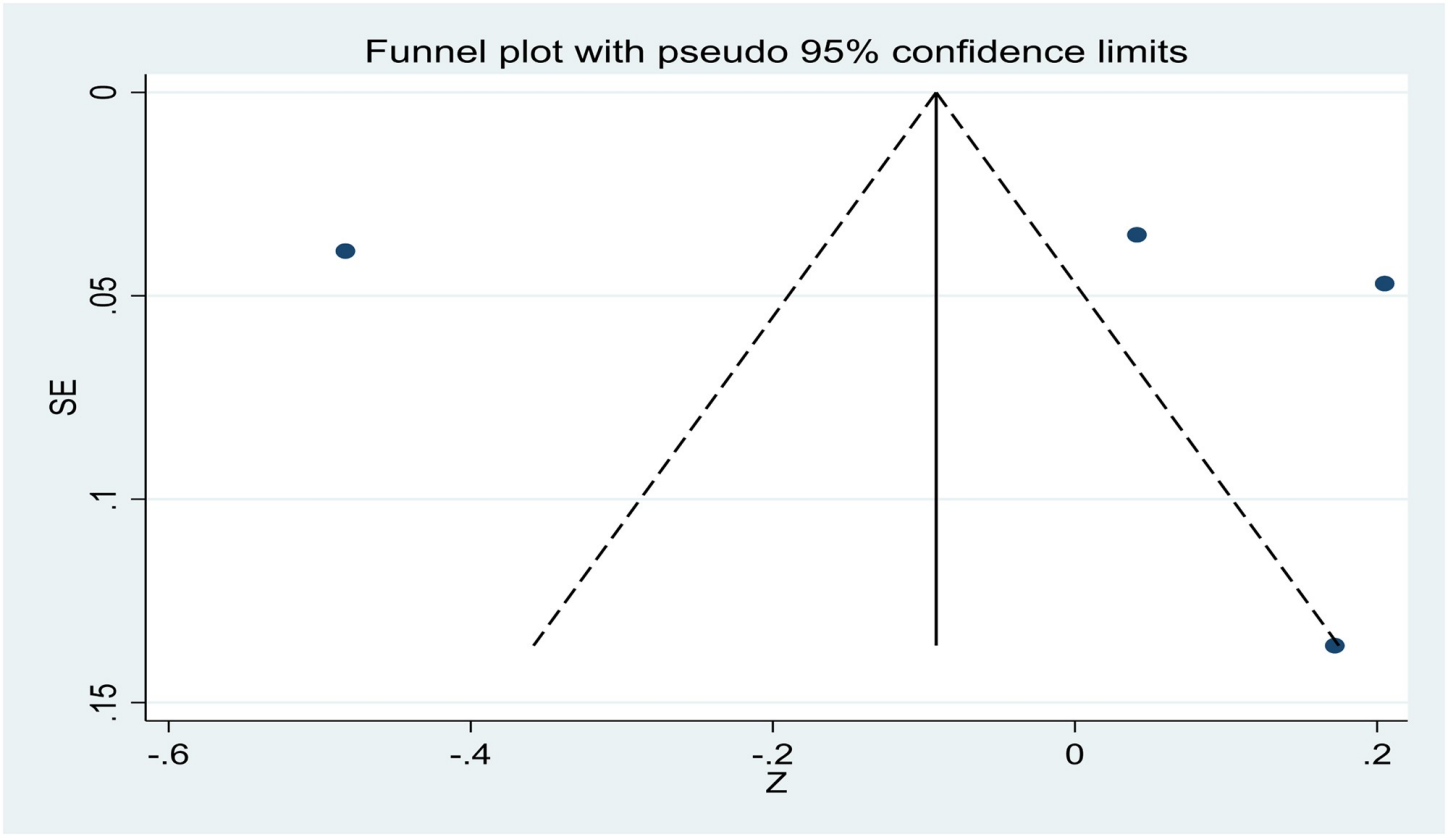
Funnel plot showing publication bias in studies investigating the relation between age and PTG.

**Fig 3:**
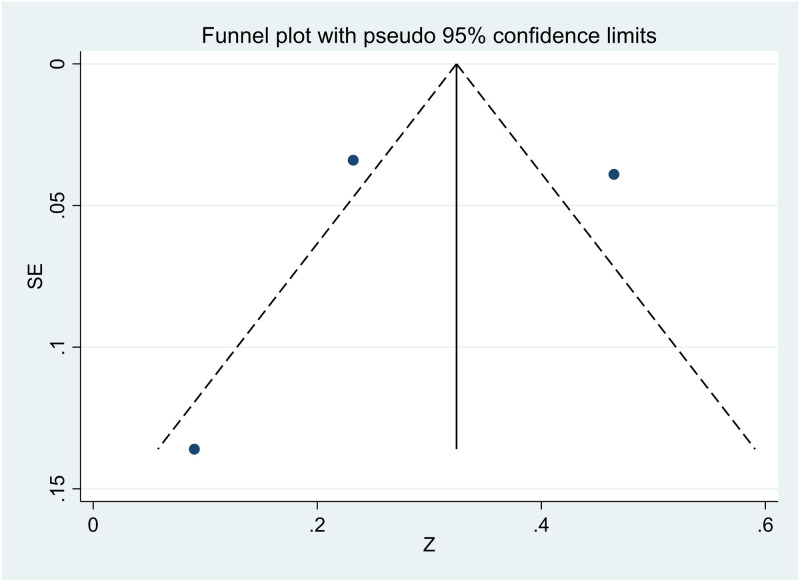
Funnel plot showing publication bias in studies investigating the relation between gender and PTG.

**Fig 4:**
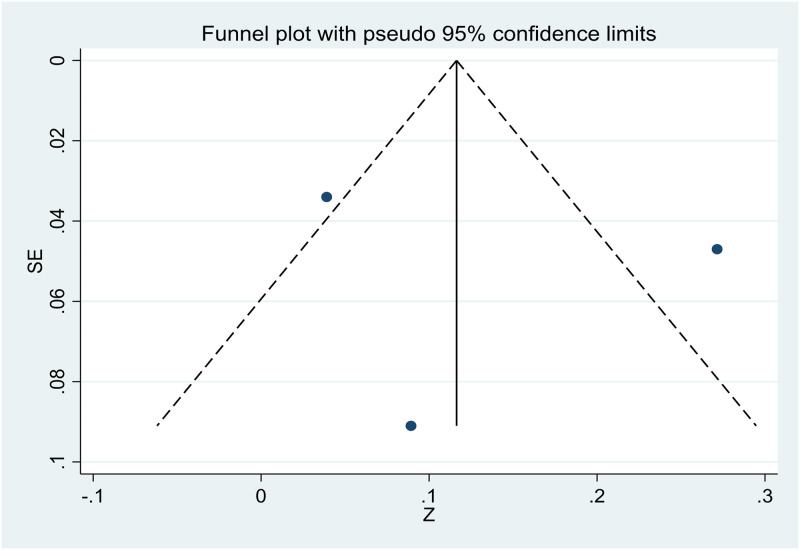
Funnel plot showing publication bias in studies investigating the relation between relationship to the deceased and PTG.

The small number of studies allowed for the assessment of publication bias only for age, gender, and relationship to the deceased. The visual inspection of the funnel plots was suggestive of publication bias due to the asymmetry in the plots (see Figs 2–4).

**Fig 5:**
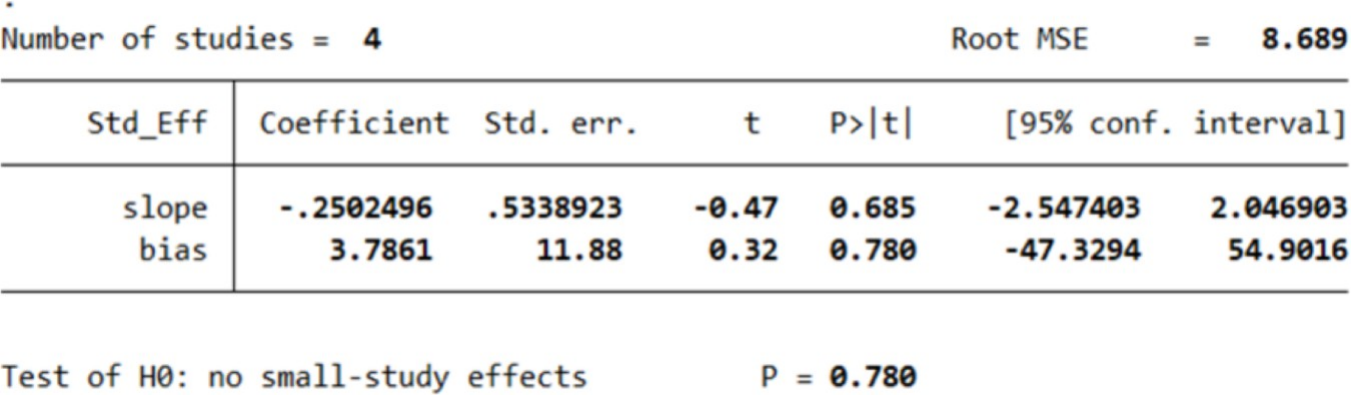
Results of Egger’s test for studies investigating the relation between age and PTG.

**Fig 6:**
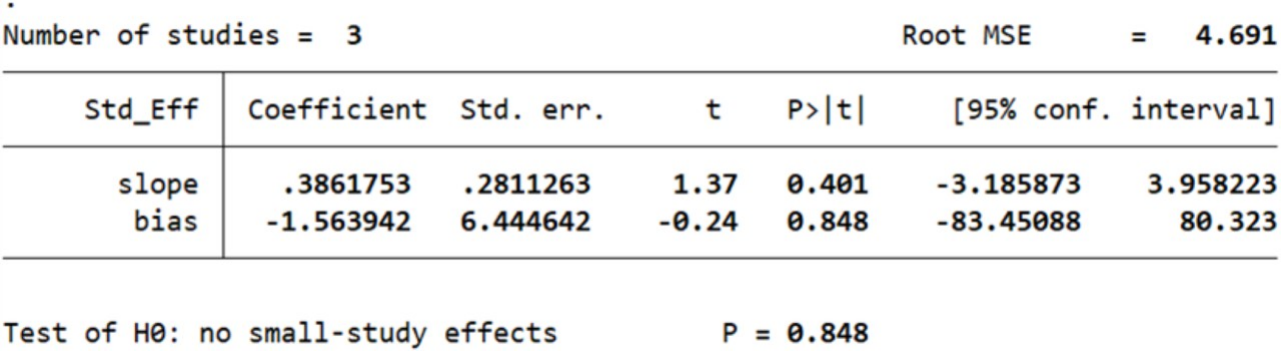
Results of Egger’s test for studies investigating the relation between gender and PTG.

**Fig 7:**
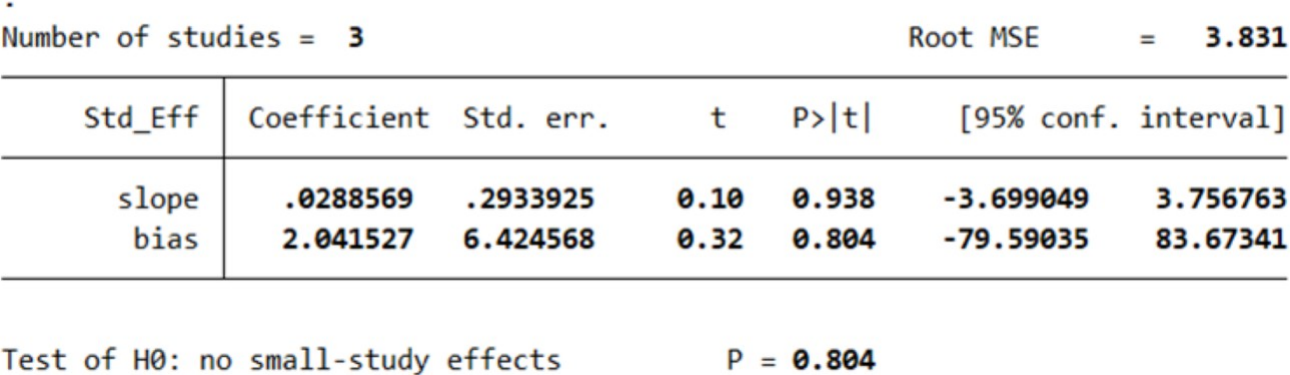
Results of Egger’s test for studies investigating the relation between relationship to the deceased and PTG.

However, Egger’s test revealed an absence of publication bias, as p > 0.05. The p values of Egger’s test for age*PTG, gender*PTG, and relationship to the deceased*PTG are 0.780 (Fig 5), 0.848 (Fig 6), and 0.804 (Fig 7), respectively. The absence of publication bias suggests that the impact of bias is trivial and the major findings are valid.

### Correlates of PTG among persons bereaved from cancer

The meta-analysis results of correlates of PTG among persons bereaved from cancer are shown in Table 5.

**Table 5:** Summary of meta-analysis results for different correlates of post-traumatic growth (PTG) among persons bereaved from cancer.

Correlate	No. of studies	Sample size	Pooled Effect Size	95% CI (UL-LL)	Heterogeneity I^2^ (%)	Model
Age	4	1981	-0.02	(-0.35–0.31)	98.1***	R
Gender	3	1577	0.27***	(0.08–0.45)	91.4***	R
Time since Death	2	1476	0.09	(-0.02–0.20)	80.3*	R
Quality of Death	2	840	0.29	(-0.01–0.54)	70.3	R
Prolonged Grief Symptoms	2	911	0.22**	(0.08–0.35)	73.3	R
Relationship to the deceased	3	1421	0.13	(-0.03–0.29)	87.6***	R
Social Support	2	505	0.13**	(0.04–0.21)	0.00	F

CI = Confidence Intervals; UL = Upper Limit; LL = Lower Limit; I^2^ = I^2^ statistic; R = Random Effects Model; F = Fixed Effects Model.

*p < 0.05,

**p < 0.01,

***p < 0.001

The review identified 17 correlates that are classified into demographic factors (age, gender, religious status, level of education), loss-related factors (time since death, quality of death, prolonged grief symptoms), interpersonal factors (relationship to the deceased, social support, attachment style, bereavement behaviours) and intrapersonal factors (resilience, coping, rumination, benevolence, meaningfulness, self-worth). The correlates analysed in the meta-analysis were age, gender, relationship to the deceased, quality of death, time since death, prolonged grief symptoms, and social support.

#### Demographic factors

**Fig 8:**
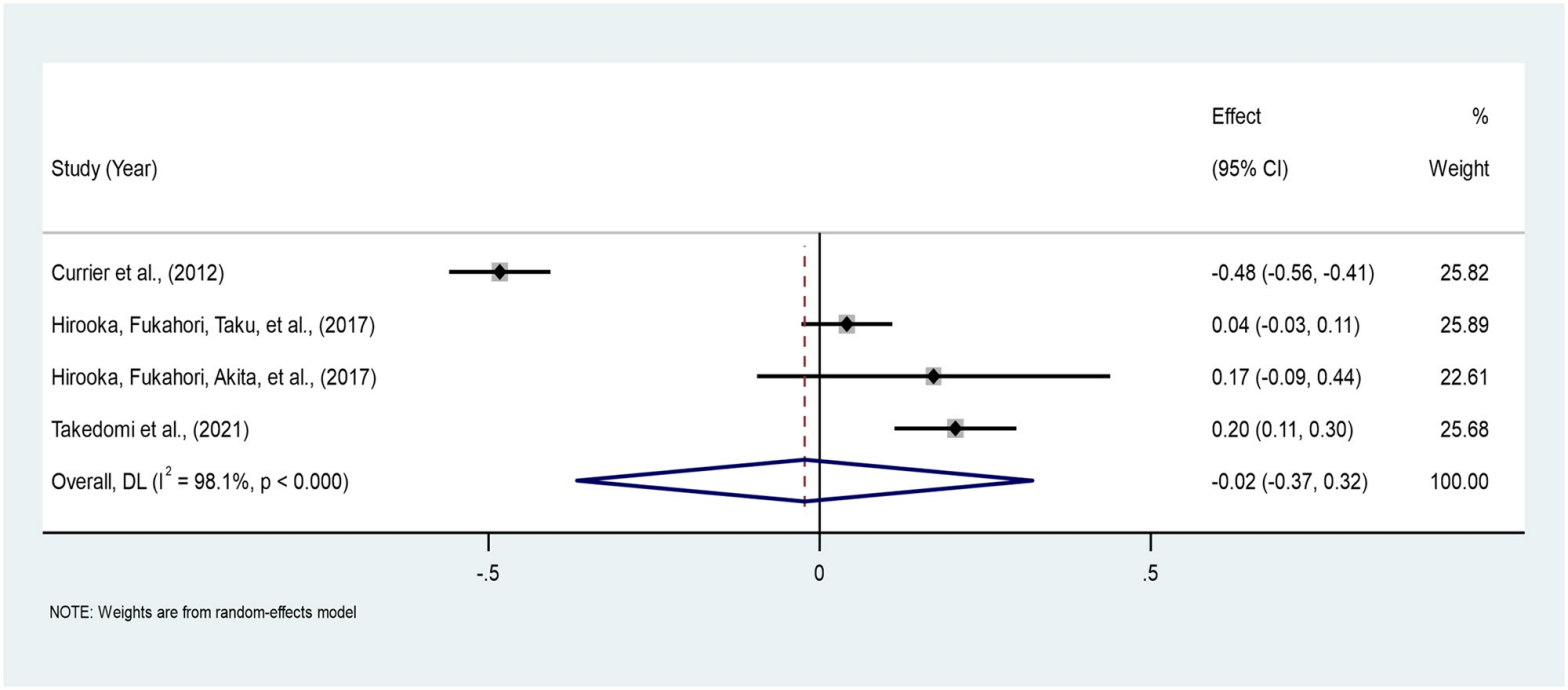
Forest plot of the meta-analysis of age*PTG among persons bereaved from cancer. Note. CI = Confidence Interval; I^2^ = I^2^ statistic for measuring heterogeneity.

*Age*. The pooled correlation between age and PTG across four studies is -0.02 (95% CI: -0.35–0.31), indicating a very weak negative correlation between age and PTG among persons bereaved from cancer. However, it is not statistically significant. The heterogeneity statistic, I^2^ shows high heterogeneity of 98.1%. The estimates ranged from -0.45 to 0.20 across the four studies [37, 40, 42, 46] (Fig 8).

**Fig 9:**
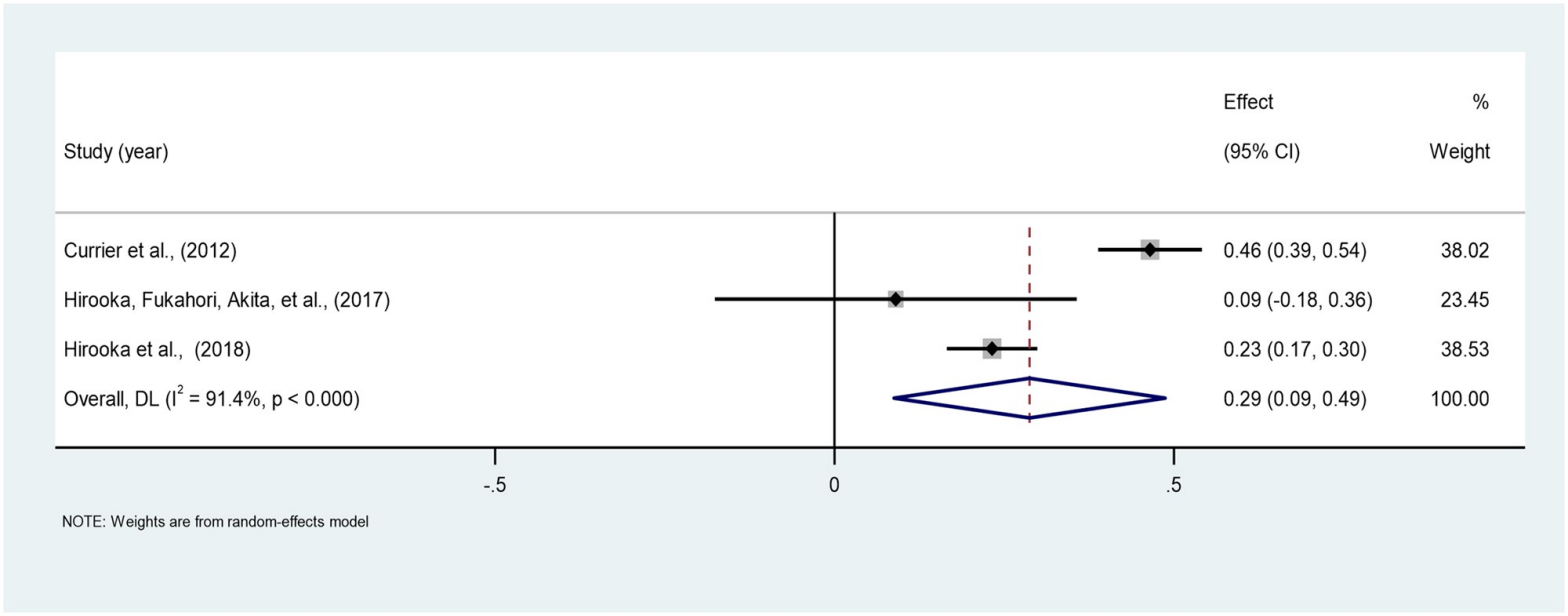
Forest plot of meta-analysis of gender*PTG among persons bereaved from cancer. Note. CI = Confidence Interval; I^2^ = I^2^ statistic for measuring heterogeneity.

*Gender*. The pooled correlation between gender and PTG across the three studies is 0.28 (95% CI: 0.08–0.45). Hence, a weak positive correlation was found between gender and PTG among persons bereaved from cancer, which was statistically significant. The heterogeneity between the studies is high, as indicated by an I^2^ value of 91.4%. The estimates ranged from 0.09 to 0.43 across the three studies [37, 40, 43] (Fig 9).

*Religious status*. Having religious beliefs is found to be associated with higher PTG [42, 43].

*Level of education*. Higher PTG was found among individuals who completed high school or university compared to those with fewer than ten years of education [48].

#### Loss-related factors

**Fig 10:**
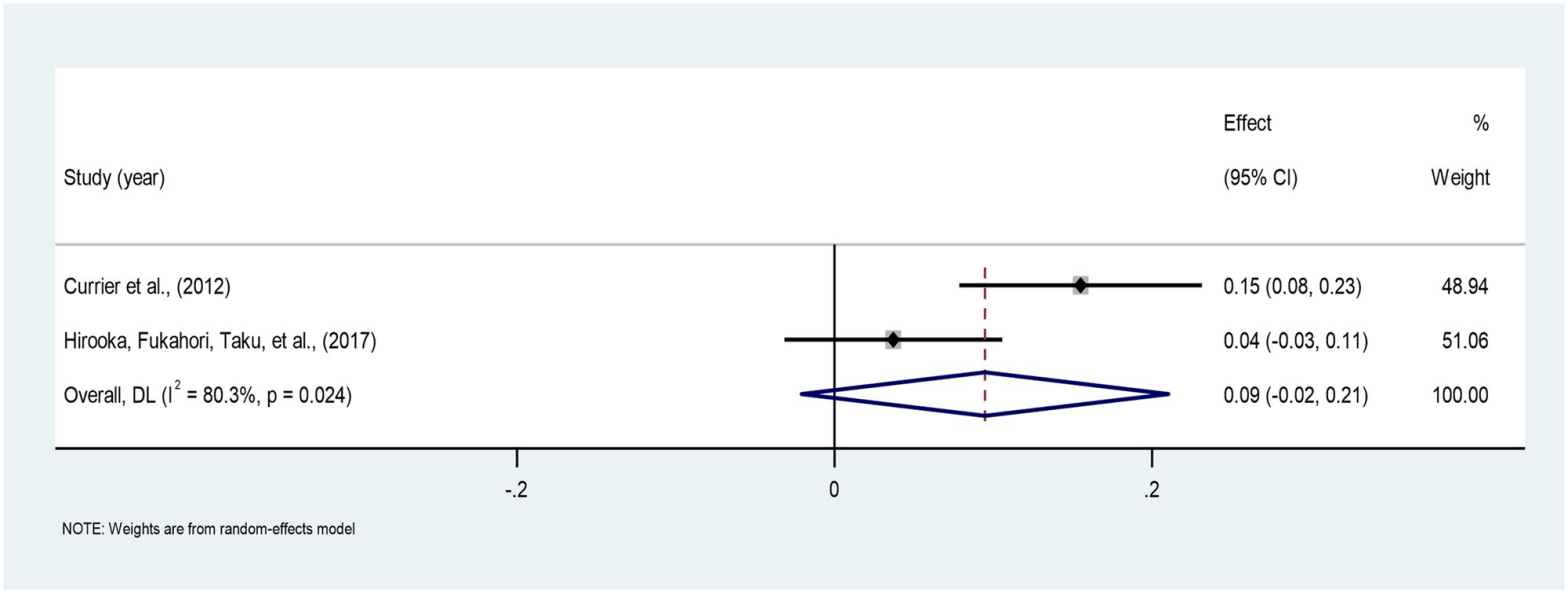
Forest plot of meta-analysis of time since death*PTG among persons bereaved from cancer. Note. CI = Confidence Interval; I^2^ = I^2^ statistic for measuring heterogeneity.

*Time since death*. The pooled correlation between time since death and PTG across the two studies is 0.09 (95% CI: -0.02–0.20), indicating a very weak positive correlation, which is not statistically significant. High heterogeneity is found (I^2^ = 80.37%). The estimates ranged from 0.04 to 0.15 across the two studies [40, 42] (Fig 10).

**Fig 11:**
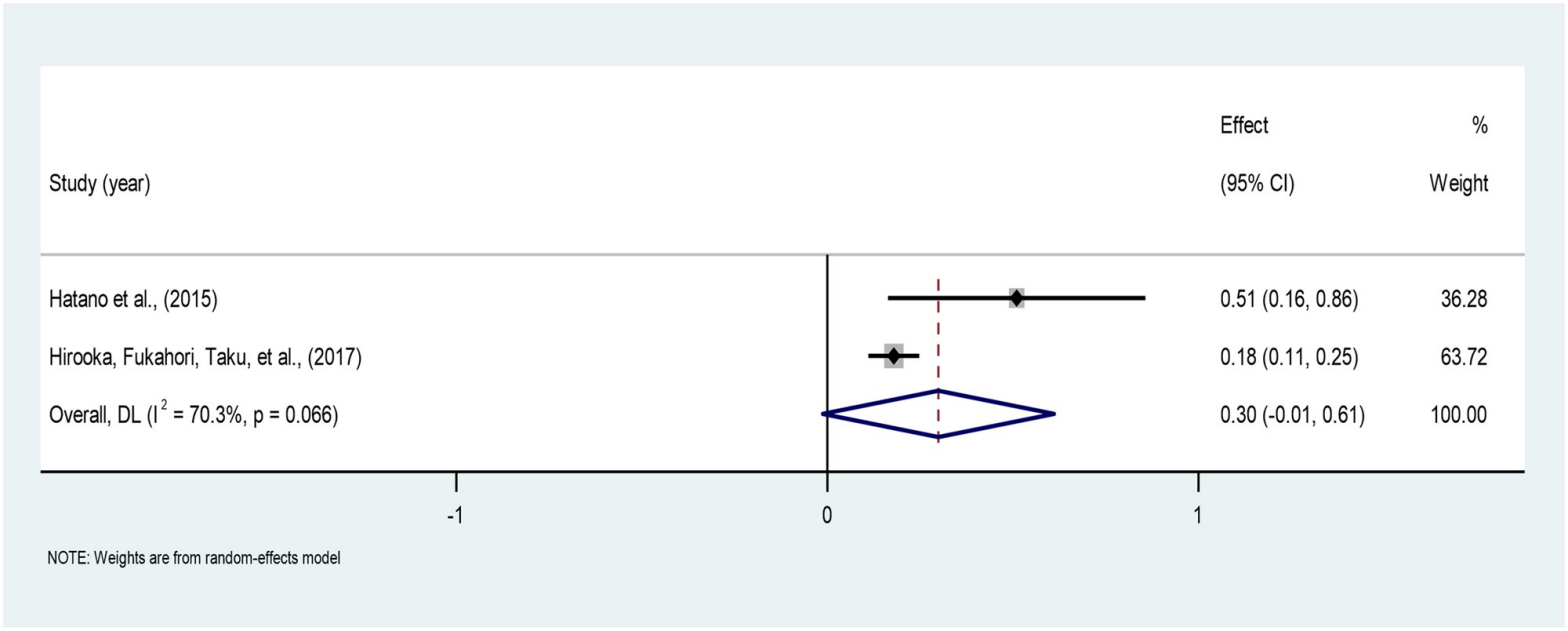
Forest plot of meta-analysis of quality of death*PTG among persons bereaved from cancer. Note. CI = Confidence Interval; I^2^ = I^2^ statistic for measuring heterogeneity.

*Quality of death*. The pooled correlation between the quality of death and PTG across the two studies is 0.29 (95% CI: -0.01–0.54,), indicating a weak positive correlation, which is not statistically significant. A moderate heterogeneity between the studies was found (I^2^ = 70.3%). The estimates ranged from 0.18 to 0.47 across the two studies [41, 42] (Fig 11).

**Fig 12:**
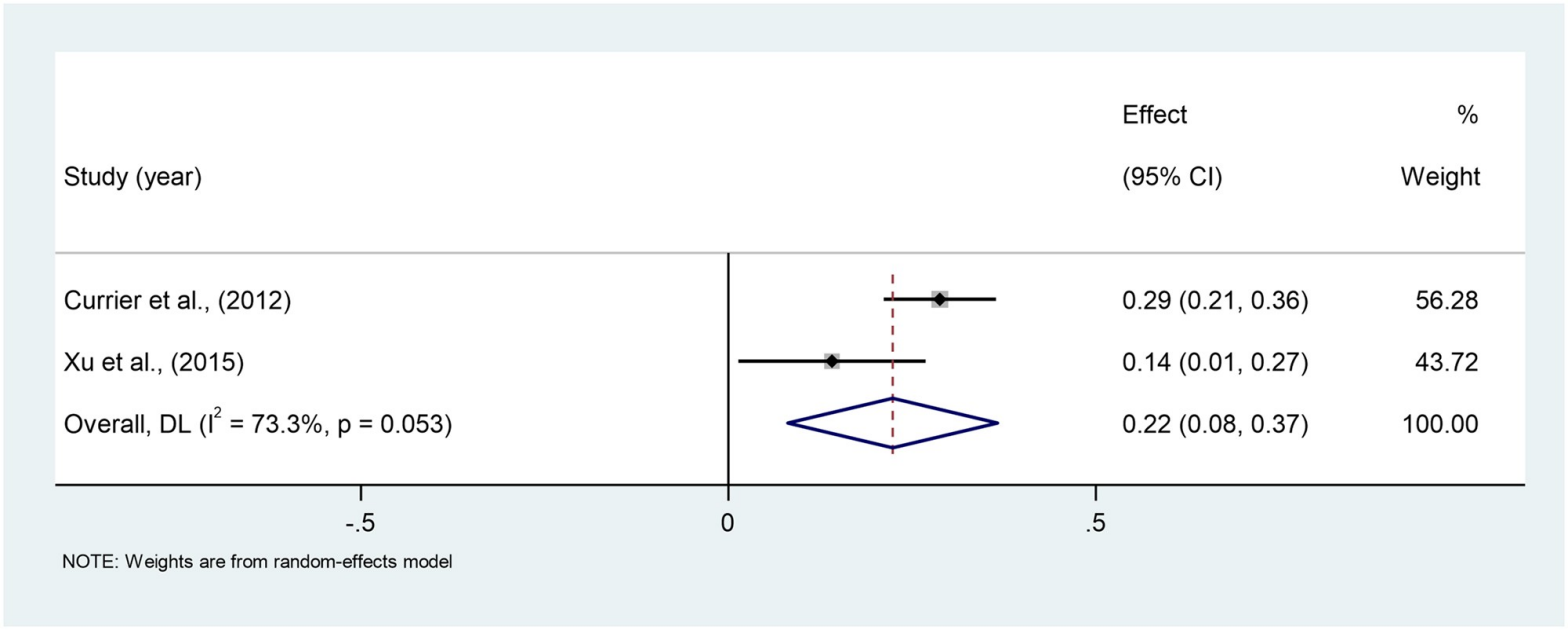
Forest plot of meta-analysis of prolonged grief symptoms*PTG among persons bereaved from cancer. Note. CI = Confidence Interval; I^2^ = I^2^ statistic for measuring heterogeneity.

*Prolonged grief (PG) symptoms*. The pooled correlation between PG Symptoms and PTG across the two studies is 0.22 (95% CI: 0.08–0.35), indicating a weak positive correlation, which was found to be statistically significant. Moderate heterogeneity is found (I^2^ = 73.3%). The estimates ranged from 0.14 to 0.28 across the two studies [40, 47] (Fig 12).

#### Interpersonal factors

**Fig 13:**
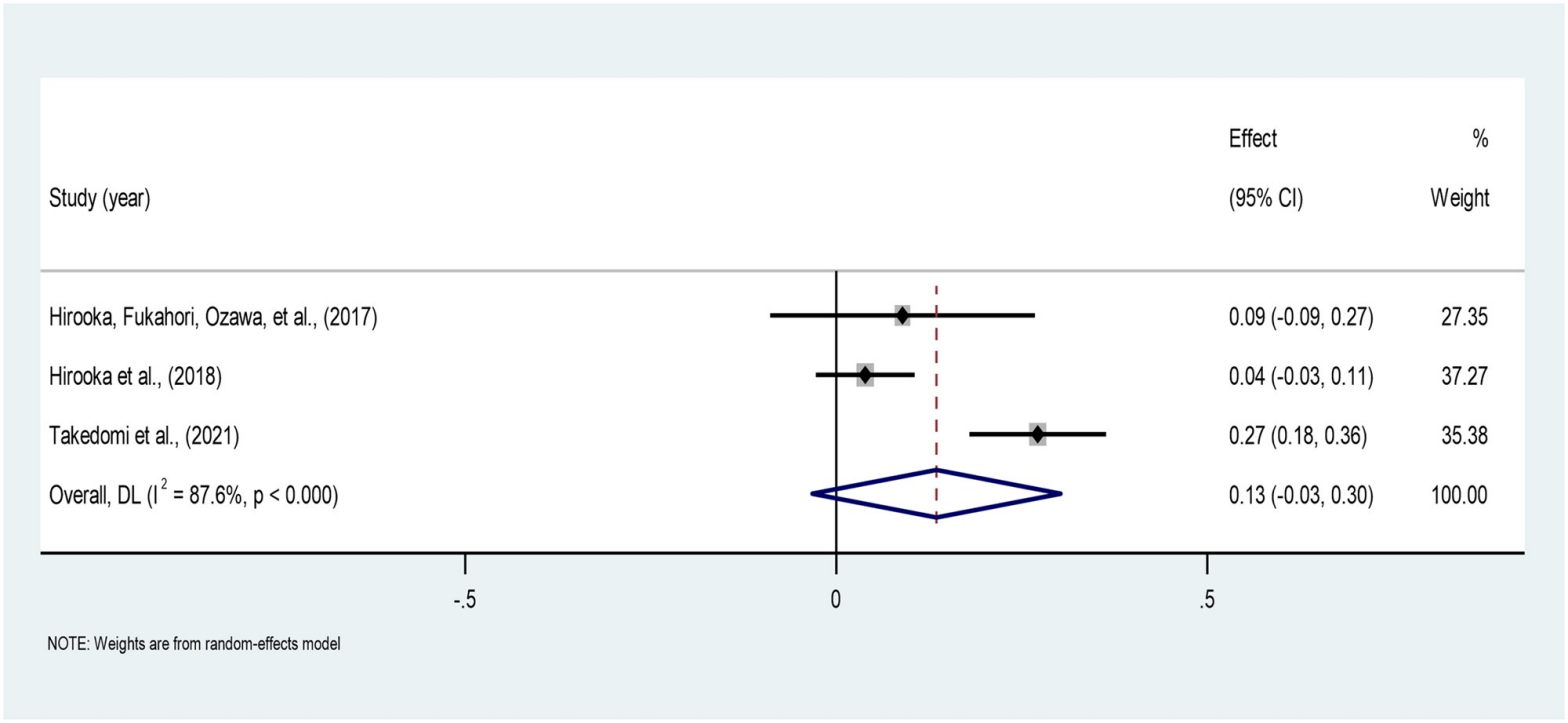
Forest plot of meta-analysis of relationship to the deceased*PTG among persons bereaved from cancer. Note. CI = Confidence Interval; I^2^ = I^2^ statistic for measuring heterogeneity.

*Relationship to the deceased*. The pooled correlation between the relationship to the deceased and PTG across the three studies is 0.13 (95% CI: -0.03–0.29), indicating a weak positive correlation which is not statistically significant. A high heterogeneity is found between the studies (I^2^ = 87.6%). The estimates ranged from 0.04 to 0.27 across the three studies [42, 43, 46] (Fig 13).

**Fig 14:**
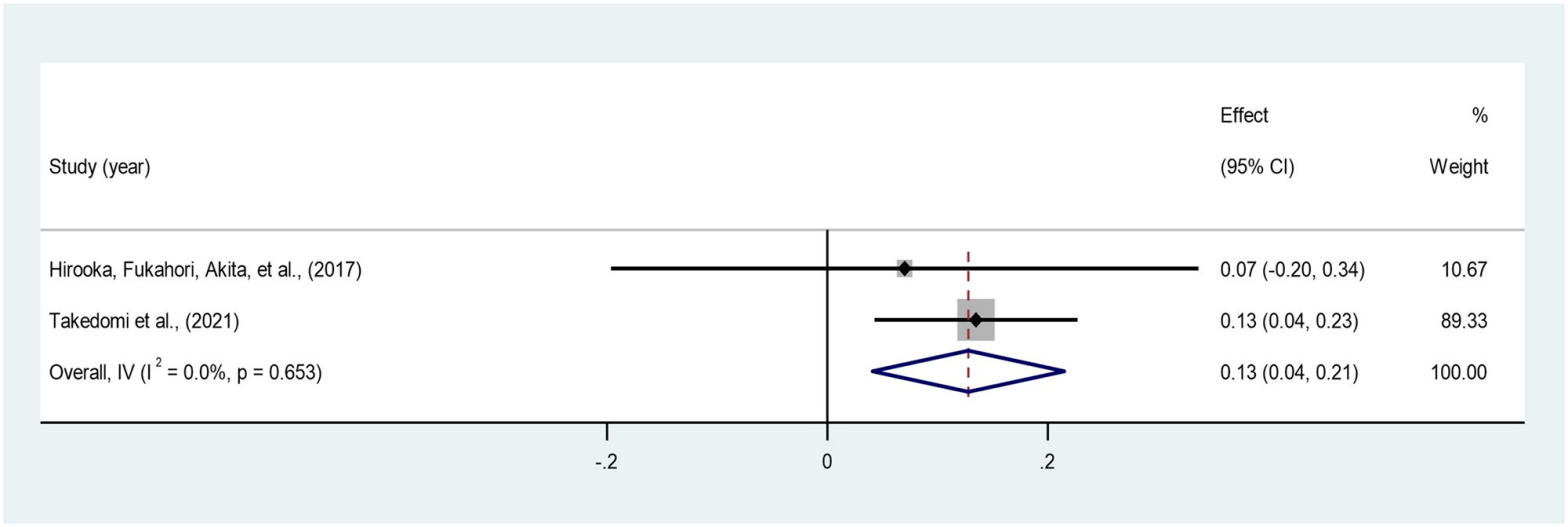
Forest plot of meta-analysis of social support*PTG among persons bereaved from cancer. Note. CI = Confidence Interval; I^2^ = I^2^ statistic for measuring heterogeneity.

*Social support*. The pooled correlation between social support and PTG across the two studies is 0.13 (95% CI: 0.04–0.21), indicating a small positive correlation, which is statistically significant. No heterogeneity was identified (I^2^ = 0.00%). The estimates ranged from 0.07 to 0.13 across the two studies [37, 46] (Fig 14).

*Attachment styles*. Xu et al. [47] reported a significant negative and positive correlation between attachment avoidance and PTG and attachment anxiety and PTG, respectively.

*Bereavement behaviours*. According to Hirooka, Fukahori, Akita, et al. [37], teenagers who engaged in certain behaviours post-bereavement, like joining hands before a memorial or altar of a deceased parent, paying a visit to a parent’s grave, and enjoying fun with friends had higher PTG scores. These behaviours encourage adolescents to reminisce about their dead parent and ruminate on the death, thereby leading to PTG.

#### Intrapersonal factors

*Resilience*. Two studies investigated the relationship between pre-loss resilience and PTG; one indicated no significant relationship [45], while the other reported a positive association [44].

*Coping*. Takedomi et al. [46] observed a significant positive relationship between emotion-focused coping and problem-focused coping with PTG. Morris et al. [38] reported that children bereaved by parental cancer engaged in low adaptive and high maladaptive coping, which was associated with lower PTG.

*Rumination*. Hirooka, Fukahori, Taku, et al. [42] reported a significant positive relationship between intrusive rumination and deliberate rumination soon after the death, recent intrusive rumination, and deliberate rumination with PTG.

*Assumptive worldviews*. A significant positive relationship was found between benevolence, self-worth, and meaningfulness (assumptive worldviews triad) with PTG [40].

## Discussion

The findings corroborate the PTG literature revealing that it does not develop as a direct consequence of trauma [13], but is also influenced by demographic, interpersonal, intrapersonal, and situational factors [30].

### Demographic factors

The meta-analysis conducted found that PTG was negatively correlated with age. It’s worth noting that only one study [40] included in the analysis reported a negative correlation between PTG and age. This was attributed to the study’s overrepresentation of younger adults. However, this study recorded a higher correlation value compared to the other three studies [37, 42, 46], which may have influenced the pooled effect size found through the meta-analysis. The review showed that most studies found higher levels of PTG in older bereaved caregivers [42, 43, 46]. This is consistent with other research suggesting that cognitive maturity is necessary to discover meaning in trauma and its consequences [52]. Additionally, many cultures consider death a taboo subject, and young people are often shielded from witnessing death and learning about how to cope with it, which could lead to lower PTG scores in younger individuals [46]. The high heterogeneity between studies regarding the age range of the sample could also be a reason for mixed findings. However, more research is needed to confirm the relationship between age and PTG among people bereaved from cancer. Nonetheless, encouraging more open discussions about death and ways to cope with it can help in the development of PTG among individuals, regardless of their age.

The meta-analysis showed a significant small positive correlation between gender and PTG. Consistent with this, a meta-analysis reported higher PTG in females as they are more likely to have ruminative thoughts than males [53]. According to the PTG model, PTG occurs when a person ruminates and tries to make sense of the past [54]. Furthermore, it is usually observed that women tend to have higher coping, social support, emotional expression, and talk about their struggles, which could contribute to higher PTG [46, 55]. Donovan et al. [48] reported that females were more likely to be the primary caregiver throughout treatment and frequently experience the daily trauma of cancer therapy. A significant precursor to PTG is a person’s ability to deal with negative ideas, feelings, and experiences [56]. Hence, their direct exposure to the pain experienced by the patients may promote PTG. Therefore, it can be understood that females experience relatively greater PTG, due to their unique adjustments and coping mechanisms. Thus, males may need more support and interventions to facilitate the development of PTG.

Further, the review highlights that having religious beliefs is associated with higher PTG. Appealing to religious beliefs or rituals might restore viewpoints by pointing to cherished goals and priorities in grieving individuals or provide purpose by pointing to the sickness as a divine plan [21]. The belief that a loved one is at peace or a belief in the afterlife may also provide strength to the bereaved [57]. Hence, encouraging and appealing to religious beliefs or tapping into spirituality may be a good way to promote PTG in bereaved individuals.

As per the level of education, it was found that people who finished high school or university showed significantly greater PTG than those with less than ten years of education. However, instead of pointing to a direct link between education level and PTG, researchers have indicated that employment tends to act as a protective factor in bereavement and that people with lower levels of education and less meaningful employment may not be able to have the opportunity for growth as their better educated, employed counterparts [48]. More research is needed to confirm this.

### Loss-related factors

A positive correlation between time since death and PTG was found, indicating that the death would have gained significance as time passed, causing the bereaved family members to experience more PTG [43]. These findings align with earlier research on bereavement and other traumatic events [58, 59]. Additionally, the immediate years following a death are marked by negative emotions and cognitions while positive changes are only achieved later on [25]. Moreover, Linley and Joseph [60] posited that PTG occurs gradually over time as it requires massive schematic changes. Hence, the need for intense cognitive processing and adaptation to achieve PTG may explain the positive correlation between time since death and PTG. However, some researchers argue that after several decades, PTG may decrease as time since death increases [28]. Thus, further research is needed to identify the specific time frame after a death that is most conducive to PTG.

On the other hand, a positive association was found between the quality of death and PTG. When bereaved family members view the death as a positive experience, they are more likely to have favourable psychological changes [41]. For family caregivers, losing someone who did not suffer and could maintain some level of quality of life was typically easier [61]. As a result, bereaved caregivers who perceived a higher quality of death have a higher PTG [42]. Thus, improving end-of-life care and the thereby quality of death may promote PTG in the cancer-bereaved.

Meta-analysis found a positive correlation between prolonged grief (PG) symptoms and PTG. Currier et al. [40] found a curvilinear link between PG symptoms and PTG, suggesting that people with in-between levels of PG symptoms had a higher chance of PTG than people with extreme levels of PG symptoms. It may be because although personal transformation frequently results from suffering [13], the possibility of positive development in the context of grief is decreased in circumstances of extreme distress due to the loved one’s passing. Therefore, the level of grief of the individuals must be taken into consideration before giving interventions to develop PTG as the distress caused by the grief may need to be addressed before any positive changes can be generated.

### Interpersonal factors

A weak positive correlation was observed between the relationship to the deceased and PTG. Specifically, two studies reported that first-degree relatives experienced higher PTG than others [39, 43]. People whose first-degree relatives died show greater grief reactions as the deceased may have been a primary attachment figure, making their death more difficult to accept [62, 63]. Conversely, two studies suggested that PTG is linked to the power of the mental relationship with the departed, not just the degree of the relationship [40, 46]. Thereby, the bereaved individual’s subjective closeness with the deceased may emerge to be a stronger predictor of PTG.

In the current review, Xu et al. [47] reported a negative association between attachment avoidance and PTG as well as a significant positive link between attachment anxiety and PTG. During bereavement, the attachment style of the individual with attachment anxiety is activated when he/she is to adapt to the separation caused and experiences high distress [64]. They may be forced to act to change the distressing circumstance as a result of the loss’s severity and significance [65].

The current meta-analysis also found a weak positive correlation between social support and PTG. A study found that the top-most unmet need of bereaved adolescents and young adults who lost either a parent or a sibling to cancer was ‘support from other young people’ [66]. It is observed that PTG occurs as an individual discloses the struggle they experienced from bereavement [46]. Thus, support groups, community intervention programs, and increasing the awareness and sensitivity of ‘*peers*’ in holding a space for the bereaved to confide in can promote the development of PTG.

### Intrapersonal factors

Participants who demonstrated resilience, positive adaptive stress coping mechanisms, and problem-focused coping showed higher levels of PTG. These findings corroborate past literature that found similar relationships in other populations [67]. However, previous research was also inconsistent, with some reporting a positive link between resilience and PTG [68], while others found that stronger resilience is linked to reduced PTG and less stress [69]. This may be because resilience may operate as a buffer against stress and assist recovery, but it does not always encourage growth. This could also mean that interventions that aim to build resilience may fall short in developing PTG in people bereaved from cancer. Thus, targeted interventions may become necessary to promote PTG.

Families adept at problem-focused coping have greater PTG rates [46]. Children bereaved by parental death due to cancer are vulnerable to maladaptive coping, including substance use, denial, self-blame, and behavioural disengagement. These are linked with lower levels of PTG and are detrimental [38]. Hirooka, Fukahori, Taku, et al. [42] reported a significant positive relationship between rumination with PTG. According to the PTG Model [13], intrusive rumination, followed by deliberate rumination, facilitates the individuals to find meaning in their situation and, thus, promotes PTG. Previous research also showed that deliberate rumination soon after the event positively affects PTG [70]. However, the relationship between rumination and PTG is not straightforward, and individual differences in coping ability exist.

Additionally, the review found that people with higher self-worth and more positive views of benevolence experienced a considerable increase in PTG. These findings are consistent with the concept proposed by Calhoun et al. [15], which contends that a crucial pathway for PTG is frequently the effective revision of core meaning systems.

### Limitations

The review found several shortcomings in the available evidence that could guide future research in the area. Due to the limited number of papers on PTG among persons bereaved from cancer, the meta-analyses could comprise only a few studies, making it challenging to draw decisive conclusions. Moreover, most of the studies were cross-sectional, making it impossible to evaluate the causation of the indicated relationships. Additionally, many studies used convenient sampling and had small sample sizes, which greatly reduced the studies’ power.

Due to the lack of available studies, many pre-planned analyses could not be performed, including sub-group analyses and meta-regression. However, heterogeneity can still be attributed to the various methodological differences between the studies, such as different populations of persons bereaved from cancer (such as bereaved caregivers, bereaved adolescents, bereaved parents, etc.), time passed since the death, sample size, geographical location, and cultural differences in the grief process. The current review also did not provide information about the impact of various correlates on the different dimensions of PTG. In addition to this, although a risk of bias assessment was done to ensure the quality of the included papers, the complete certainty assessment to assess the confidence in the source of evidence was not performed. The publication bias assessment and the meta-analyses were done using a very small number of studies. Thus, it is recommended that the findings be interpreted with caution.

### Implications

#### Implications for practice

The current review underscores how understanding the various correlates of PTG can enable palliative care nurses, doctors, and therapists to identify vulnerable individuals and those with the potential to develop PTG. This knowledge can facilitate the provision of more targeted and personalised interventions that mitigate maladaptive grief reactions and promote growth after the loss. Additionally, healthcare professionals can benefit from education programs designed to enhance their understanding of PTG, enabling them to recognise signs and factors that influence it. By doing so, the quality of support provided to the bereaved can be improved. Integrating the existing support systems like family, friends, religious communities etc in the care plan can enrich the overall support network for bereaved individuals and contribute to PTG.

#### Implications for healthcare providers and policymakers

Policymakers can consider incorporating PTG-focused elements into grief support protocols and community-based initiatives to promote social support and address interpersonal factors that contribute to PTG. Collaboration with community organisations and religious institutions needs to be developed, and resources should be allocated to support groups, counselling services, and educational programs that specifically target factors influencing PTG. It is important to note that both end-of-life care at home and hospice care are expensive and underdeveloped in many countries. Hence, improving opportunities for end-of-life care at home and hospice care can help terminally ill patients and caregivers throughout their journey and ensure PTG among the bereaved.

#### Recommendations for future research

Since a very small number of studies explored PTG among persons bereaved from cancer, there is a great scope for various research endeavours in this area. The present literature has yet to explore the impact of various factors such as socioeconomic status, optimism, empathy, other trauma, communication styles, family dynamics, professional support, type of cancer, and age at death on post-traumatic growth (PTG) among cancer-bereaved individuals. To achieve a comprehensive understanding of the factors influencing PTG in this population, future studies can employ comparative methods that explore PTG in various populations. More longitudinal studies are needed to understand the dynamic nature of PTG and to examine how various factors influencing PTG evolve so that interventions designed to meet the changing needs of the bereaved can be developed.

It would be valuable for future research to examine how cultural differences influence the experience of PTG. Specifically, the impact of cultural rituals, beliefs, and support systems on the development of PTG should be investigated. Considering the influence of culture on PTG, there may also be a need for reconsidering the dimensions of PTG. Moreover, it is worthwhile to explore how PTG affects the mental health, well-being, and quality of life of individuals who are grieving. Additionally, studies should explore how regional or geographical differences may affect PTG.

An important area for future research is investigating the mechanisms through which specific factors influence PTG. Future studies can also evaluate the efficacy of various interventions in promoting PTG among individuals who have lost a loved one to cancer. Furthermore, exploring which interventions are most appropriate for each person based on their unique needs and circumstances is also an important avenue for future research.

## Conclusion

PTG experienced by the bereaved who lost a loved one to cancer is a positive development that can help them find the ‘*silver lining’* in the trauma and emerge as a transformed human being. The current review identified several demographics, loss-related, interpersonal and intrapersonal factors that are associated with PTG among people bereaved from cancer, revealing the possibility of a complex interplay among these factors. These findings can aid healthcare professionals by enhancing their understanding of PTG, resulting in improved tailoring of interventions and better care for bereaved individuals. Integrating the existing support systems like family, friends, religious communities etc in the care plan can enrich the overall support network for bereaved individuals. Improving opportunities for end-of-life care at home and hospice care and making relevant policy changes can promote PTG. However, due to limitations such as the small number of studies, small sample sizes and methodological differences, the results must be interpreted with caution.

The review highlighted the need for further exploration of mixed findings, stressing the importance of considering diverse cultural factors. Also, future research has to focus on a comprehensive exploration of different correlates of PTG and their interactions, longitudinal studies, and cultural influences. The limited number of studies on PTG among persons bereaved from cancer suggests the need for increased attention, understanding, and conceptualisation of PTG in this significant population. Nonetheless, this review improves the understanding of PTG among persons bereaved from cancer, providing a foundation for future efforts to improve support mechanisms for those navigating the challenging journey of grief.

## Supporting information

S1 Checklist PRISMA checklist. (DOCX)
