# Correction: Correlates of post-traumatic growth among persons bereaved from cancer: A systematic review and meta-analysis

**DOI:** 10.1371/journal.pone.0360071

**Published:** 2026-10-06

**Authors:** K. N. V. Sri Lakshmi, Eslavath Rajkumar, Aswathy Gopi, P. V. Hareesh, John Romate, R. Lakshmi, John Abraham

The first author’s name is written incorrectly. The correct name is: K. N. V. Sri Lakshmi. The correct citation is: Sri Lakshmi KNV, Rajkumar E, Gopi A, Hareesh PV, Romate J, Lakshmi R, et al. (2024) Correlates of post-traumatic growth among persons bereaved from cancer: A systematic review and meta-analysis. PLoS ONE 19(3): e0300291. https://doi.org/10.1371/journal.pone.0300291.
